# The Anti-HIV Actions of 7- and 10-Substituted Camptothecins

**DOI:** 10.3390/molecules15010138

**Published:** 2009-12-31

**Authors:** Yu-Ye Li, Shi-Wu Chen, Liu-Meng Yang, Rui-Rui Wang, Wei Pang, Yong-Tang Zheng

**Affiliations:** 1 Key Laboratory of Animal Models and Human Disease Mechanisms of Chinese Academy of Sciences & Yunnan Province, Kunming Institute of Zoology, Chinese Academy of Sciences, Kunming, Yunnan 650223, China; 2 School of Pharmacy, Lanzhou University, Lanzhou 730000, China; 3 Graduate School of the Chinese Academy of Sciences, Beijing 100039, China; 4 The First Affiliated Hospital of Kunming Medical College, Kunming, Yunnan 650032, China; 5 Yunnan Institute of Dermatology & Venereology, Kunming, Yunnan 650032, China

**Keywords:** camptothecin, anti-HIV agents, HIV

## Abstract

Camptothecin (CPT), a traditional anti-tumor drug, has been shown to possess anti-HIV-1 activity. To increase the antiviral potency, the anti-HIV activities of two CPT derivatives, 10-hydroxy-CPT and 7-hydroxymethyl-CPT, were evaluated *in vitro*. The therapy index (TI) of CPT, 10-hydroxy-CPT and 7-hydroxymethyl-CPT against HIV-1_IIIB_ in C8166 were 24.2, 4.2 and 198.1, and against clinical isolated strain HIV-1_KM018_ in PBMC were 10.3, 3.5 and 66.0, respectively. While the TI of CPT, 10-hydroxy-CPT and 7-hydroxymethyl-CPT against HIV-2_CBL-20_ were 34.5, 10.7 and 317.0, respectively, and the TI of the three compounds against HIV-2_ROD_ showed the similar values. However, when the antiviral mechanisms were considered, we found there was no inhibition of 7-hydroxymethyl-CPT on viral cell-to-cell transmission, and was no inhibition on reverse transcriptase, protease or integrase in cell-free systems. 7-Hydroxymethyl-CPT showed no selective killing of chronically infected cells after 3 days of incubation. In conclusion, 7-hydroxymethyl-CPT showed more potent anti-HIV activity, while 10-hydroxy-CPT had less efficient activity, compared with the parent CPT. Though the antiviral mechanisms remain to be further elucidated; the modification of -OH residues at C-7 of CPT could enhance the antiviral activity, while of -OH residues at C-10 of CPT had decreased the antiviral activity, which provides the preliminary modification strategy for anti-viral activities enhancement of this compound.

## 1. Introduction

HIV/AIDS is still a global public health problem. The U.S. FDA hitherto has approved a number of HIV inhibitors for clinical usage to fight against HIV infection based on intervention in the viral life cycle. The usage of combinations of chemical drugs, the so called highly active antiretroviral therapy (HAART), has been shown especially to improve the life quality and life span of the patients with HIV/AIDS in the past decades [1]. However, HAART is not able to eradicate latently infected HIV-1 in patients yet; additionally, drug resistance, severe side effects, and the high costs of the current clinically available chemical drugs have hindered the development of antiretroviral therapy [2]. Therefore, the continuous development of new kinds of antiviral agents, such as the lead compounds isolated from natural species which have been proved to possess the priorities of non-inducible of drug resistance, lower cytotoxicity and costs, are extremely necessary [3].

**Figure 1:**
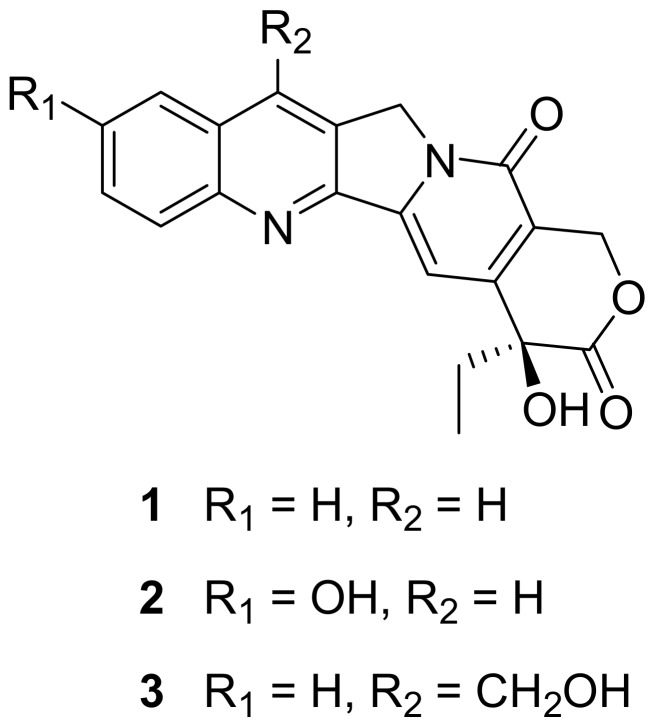
Structures of Camptothecin (**1**, MW 348) and its analogues (**2**, MW 365; **3**, MW 378).

Camptothecin (CPT, **1**, Figure 1) is a traditional anti-tumor drug, which was first isolated from the Chinese tree *Camptotheca acuminata* by Wall *et al.* in 1966 [4]. CPT and its derivatives might effect their anti-tumor activity via the repression of the activity of topoisomerase I [5], or via the induction of apoptosis in tumor cells [6]. CPT had shown to weakly inhibit replication of the equine anemia virus and HIV [7], while a derivative, namely 9-nitrocamptothecin (9NC), showed inhibition of HIV-1 replication in freshly infected U937 monocytoid cells [8]. Topotecan, a semisynthetic analogue of **1**, was efficient in treatment of AIDS-related progressive multifocal leukoencephalopathy [9]. In this study, we investigated *in vitro* anti-HIV activities (including HIV-1 and HIV-2) of two CPT analogues, named as 10-hydroxy-CPT (**2**) and 7-hydroxymethyl-CPT (**3**) (Figure 1).

## 2. Results and Discussion

### 2.1. Cytotoxicities of Camptothecin and Its Derivates

**Figure 2:**
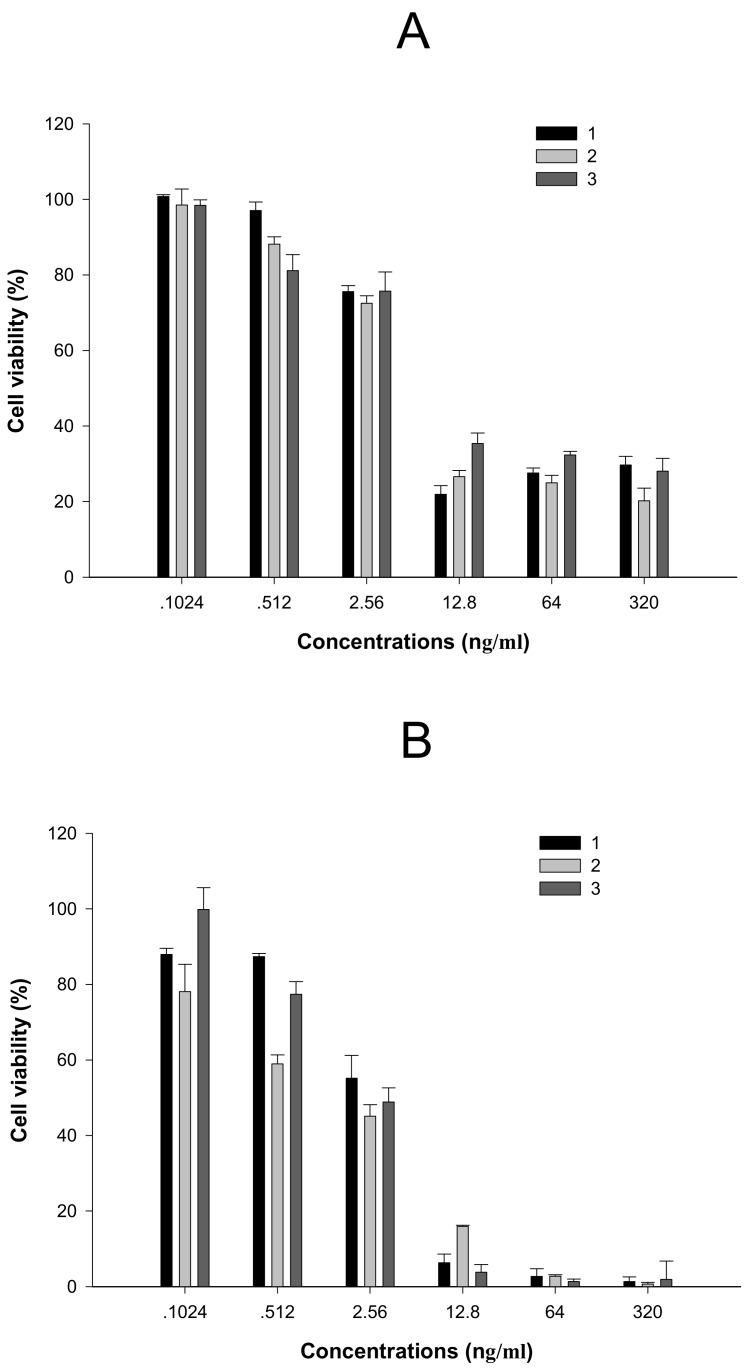
Cytotoxities of compounds 1-3 on C8166 (A) and PBMC (B).

Compounds **2**, **3** showed similar cytotoxicities towards T cell line C8166 (Figure 2A) and PBMC (Figure 2B), compared with CPT. Data are expressed as means±SD. The CC_50_ values of **1**, **2** and **3** on C8166 were 137.8, 140.9 and 158.5 ng/mL, and on PBMC they were 75.9, 36.3 and 60.0 ng/mL, respectively (Table 1). All three compounds exhibited lower cell survival at a concentration of 320 ng/mL or higher, therefore those concentrations lower than 320 ng/mL, in which higher cell viability could be maintained, were mainly used for the antiviral activity assays in this study.

### 2.2. The Anti-HIV-1 Activities

**Figure 3:**
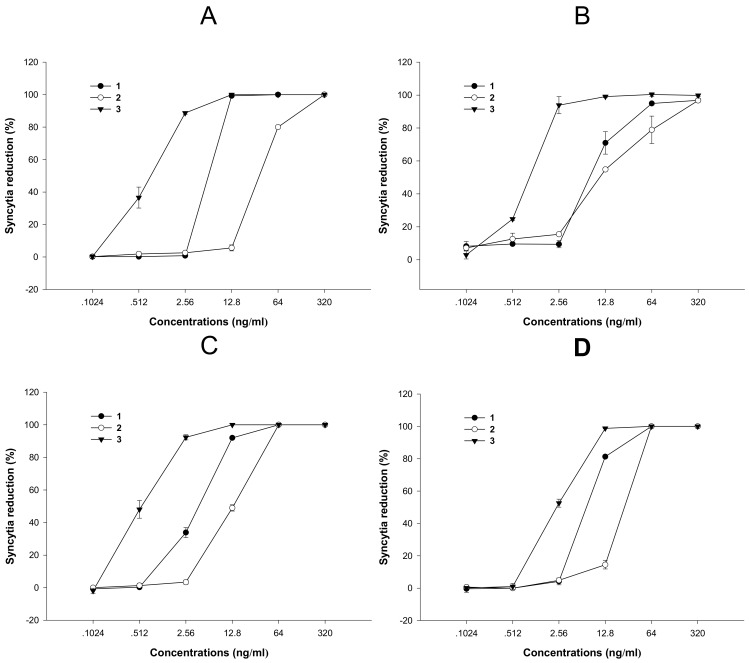
The anti-HIV activities of compounds **1**-**3**. (A) The CPE Inhibition induced by HIV-1_IIIB_ on C8166 cells; (B) The replication inhibition of clinically isolated strain HIV-1_KM018_ in PBMC by p24 antigen quantification; (C) The CPE inhibition induced by HIV-2_CBL-20_ on C8166 cells; (D) The CPE inhibition induced by HIV-2_ROD_ on C8166 cells.

The antiviral activities were mainly evaluated by the inhibition of CPE formation and HIV-1 gag protein p24 antigen production. Compounds **1**, **2** and **3** exhibited inhibition of CPE formation induced by HIV-1_IIIB_ on C8166 cells with EC_50_ values of 5.7, 33.4 and 0.8 ng/mL, respectively (Figure 3A, Table 1), so the corresponding therapeutic index (TI) of compounds **1**, **2** and **3** against HIV-1_IIIB_ were 24.2, 4.2 and 198.1, respectively (Table 1). The potential inhibition on viral replication also was assessed by measuring expression of HIV-1 p24 antigen. Compounds **1**, **2** and **3** inhibited clinically isolated virus HIV-1_KM018_ replication in PBMC with EC_50_ values of 7.4, 10.5 and 0.9 ng/mL, respectively (Figure 3B, Table 1). Therefore the TI values of compounds **1**, **2** and **3** against HIV-1_KM018_ were 10.3, 3.5 and 66.0, respectively (Table 1).

### 2.3. Anti-HIV-2 Activities

The potential inhibitions of HIV-2 by CPT and its derivates **2** and **3** were also evaluated. CPT, 10-hydroxy-CPT and 7-hydroxymethyl-CPT inhibited C8166 CPE formation induced by HIV-2_CBL-20_ replication with EC_50_ values of 4.0, 13.2 and 0.5 ng/mL, respectively (Figure 3C), the TI values of CPT, 10-hydroxy-CPT and 7-hydroxymethyl-CPT against HIV-2_CBL-20_ were 34.5, 10.7 and 317.0, respectively (Table 1). CPT, 10-hydroxy-CPT and 7-hydroxymethyl-CPT could also inhibit C8166 CPE formation induced by HIV-2_ROD_, with EC_50_ values of 6.7, 25.0 and 2.4 ng/mL, respectively (Figure 3D), and the TI of CPT, 10-hydroxy-CPT and 7-hydroxymethyl-CPT against HIV-2_ROD_ were 20.6, 5.6 and 66.0, respectively (Table 1).

### 2.4. Mechanistic Clarification of Antiviral Activities

7-Hydroxymethyl-CPT, which possesses the best anti-HIV activities amongst the three compounds, was selected as the representative to address antiviral mechanism. The potential inhibition of cell-to-cell transmission of HIV was first tested. Chronically infected H9/HIV-1_IIIB_ cells were co-cultured with uninfected C8166 cells in presence of different concentrations of 7-hydroxymethyl-CPT, however, no inhibition of cell-to-cell transmission of HIV-1_IIIB_ has been observed, even at a dosage of 2000 ng/mL.

The potential inhibition assay on HIV reverse transcriptase and protease also were followed. While 7-hydroxymethyl-CPT could not inhibit the activity of recombinant reverse transcriptase and protease even at the dosages of 17,000 ng/mL and 40,000 ng/mL, respectively, it bound HIV-1 integrase with a *Kd* value of 24,570 ng/mL (Table 1).

**Figure 4:**
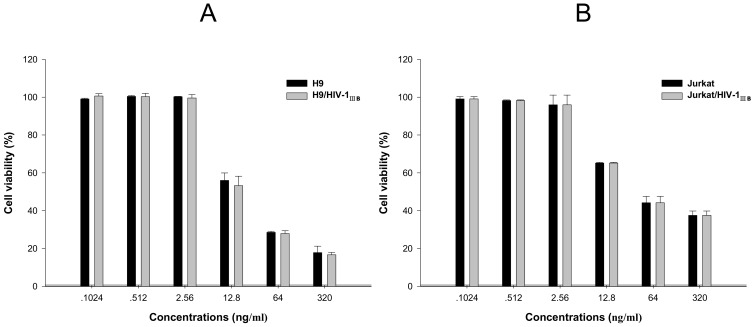
Comparison of the cytotoxicities of compound **3** on HIV-1_IIIB_ chronically infected cells and uninfected cells. (A) The cytotoxicity on H9 and H9/HIV-1_IIIB_ cells. (B) the cytotoxicity on Jurkat and Jurkat/HIV-1_IIIB_ cells.

Additionally, there was no selective killing on HIV infected cell after three days of incubation, because 7-hydroxymethyl-CPT showed similar cytotoxicity on chronically infected H9/HIV-1_IIIB_ cells compared with uninfected H9 cells (Figure 4A), and on acutely infected Jurkat/HIV-1_IIIB_ cells compared with uninfected Jurkat cells (Figure 4B).

### 2.5. Discussion

Although clinically effective when used in combination, none of the currently available anti-HIV drugs or regimens represents ideal therapies, due to drug-related side effects, inconvenient dosing requirements, and the emergence of drug resistant virus [10]. Thus, development of new antiviral agents with novel mechanisms is necessary. CPT and its derivatives provide a wide spectrum of biological and pharmacological properties [11]. They may offer more opportunities to find anti-HIV drugs or lead anti-microbial compounds.

In order to increase the antiviral potency, two CPT derivatives **2** and **3** have been synthesized, and their anti-HIV activities were further evaluated. 7-Hydroxymethyl-CPT showed more efficient anti-HIV-1 activity than CPT, while 10-hydroxy-CPT had less efficiency. The TI values (HIV-1_IIIB_ in C8166) of 10-hydroxy-CPT and 7-hydroxymethyl-CPT were 4.2 and 198.1, respectively. Similar results were obtained when the antiviral activities on a clinical isolate HIV-1_KM018_ strain were tested. 7-Hydroxymethyl-CPT showed more potent anti-HIV-2 activity than CPT, and 10-hydroxy-CPT had less efficient anti-HIV-2 activity than CPT. Thus the modification of -OH residues at the C-7 site of CPT could enhance the antiviral activity, while the modification of -OH residues at C-10 site of CPT had decreased the antiviral activity.

The exact mechanism of the inhibition of HIV replication by CPT derivatives remains an enigma and we have tried to elucidate it in the current study. 7-Hydroxymethyl-CPT showed no inhibition of viral cell-to-cell transmission; 7-hydroxymethyl-CPT did not show any inhibition of reverse transcriptase, protease or integrase in the cell-free enzymatic assay system; the acute cytotoxicity of 7-hydroxymethyl-CPT against chronically infected cells and uninfected cells was similar, indicating there is no selective killing of HIV-1 chronically infected cells. 9-Nitrocamptothecin (9NC) is a derivative of camptothecin used as potential HIV therapy. It inhibited tumor recrosis factor-mediated activation of HIV-1 and enhances apoptosis in a latently infected T cell clone [12]. 9NC reduced HIV-1 replication by inducing selective apoptosis of HIV infected cells [11]. The antiviral mechanism of 7-hydroxymethyl-CPT needs to be further investigated. Cell cycle profiles, apoptosis and other viral life cycle targets will be the subject of the further work.

## 3. Experimental

### 3.1. Reagents

AZT was purchased from Sigma (Steinheim, Germany). Horseradish peroxidase (HRP) labeled goat anti-human IgG was purchased from Sino-America Biotechnology Company (Beijing, China). P5F1, monoclonal antibody (McAb) against HIV-1 p24 was prepared by our laboratory. Human polyclonal anti-HIV-1 serum was kindly donated by Dr. Hiroo Hoshino of the Gunma University School of Medicine (Maebashi, Japan).

### 3.2. Chemistry

CPT was isolated from a Chinese medicinal plant *Camptotheca acuminata* and served as the starting material for the preparation of the derivatives 10-hydroxy-CPT and 7-hydroxymethyl-CPT. Briefly, 10-hydroxy-CPT was prepared from CPT by a reduction-oxidation sequence [13], and 7-hydroxymethyl-CPT was obtained by reacting CPT with FeSO_4_, methanol, H_2_SO_4_, and H_2_O_2_ [14].

### 3.3. Cells and Virus

Cell lines (C8166, H9, H9/HIV-1_IIIB_, Jurkat, Jurkat/HIV-1_IIIB_) were maintained in RPMI-1640 supplemented with 10% heat-inactivated newborn calf serum (Gibco, Auckland, New Zealand). The cells used in all experiments were in log-phase growth. PBMC from healthy donors were isolated by Ficoll-Hypaque centrifugation, then incubated in complete medium containing 5μg/mL phytohemagglutinin (PHA) (Sigma) and 50 U/mL human recombinant IL-2 for 72 h prior to use for antiviral assays. The laboratory-derived viruses (HIV-1_IIIB_, HIV-2_ROD_, HIV-2_CBL-20_) were obtained from the Medical Research Council (MRC), AIDS Reagent Project (UK) and the NIH AIDS Research and Reference Reagent Program (USA). The clinically isolated HIV-1_KM018_ was obtained from a naive HIV-1-infected individual in Yunnan Province, China, as described [15]. The 50% HIV-1 tissue culture infectious dose (TCID_50_) was determined and calculated by the Reed and Muench method. Virus stocks were stored in aliquots at −70 °C. 

### 3.4. MTT-Based Cytotoxicity Assay

Cellular toxicity of compounds **1**–**3** was assessed by the MTT method, as described previously [16]. Briefly, cells were seeded on a microtiter plate in the absence or presence of various concentrations of compounds in triplicate and incubated at 37 °C in a humid atmosphere of 5% CO_2_ for three days. Twenty microliters of MTT reagent (5 mg/mL in PBS) was added to each well, then incubated at 37 °C for 4 h. One hundred microliters of 50% DMF-20% SDS was then added. After the formazan was dissolved completely, the plates were read on a Bio-Tek ELx 800 ELISA reader at 595 nm/630 nm. The results were shown as absorbance values, and the CC_50_ was calculated. 

### 3.5. Syncytia Assay

In the presence of 100 μL of various concentrations of the tested compound, C8166 cells (4 × 10^5^cells/mL) were infected with virus (HIV-1_IIIB,_ HIV-2_CBL-20_ or HIV-2_ROD_) at a multiplicity of infection (M.O.I.) of 0.06. The final volume per well was 200 μL. Control assays were performed without the testing compounds in HIV-1 _IIIB_ infected and uninfected cultures. AZT was included as positive control. After three days of culture, the cytopathic effect (CPE) was measured by counting the number of syncytia in each well under an inverted microscope. EC_50_ was then calculated [17]. 

### 3.6. Inhibition of HIV-1 p24 Antigen Production in PBMC

Adequate numbers of PHA activated normal PBMC were incubated with HIV-1_KM018_ (M.O.I. = 0.03) in presence of varying concentrations of the compound. After 3 h of virus adsorption, the cells were washed twice with PBS and incubated with or without various concentration of compound in culture medium supplemented with 50 U/mL human recombinant IL-2 at 1.2 × 10^6^ cells/mL for seven days. Half of the medium was changed with corresponding compound concentrations on the third day. At seven days post-infection, HIV-1 p24 antigen in cell-free culture supernatants was analyzed by ELSIA. The percent inhibition of HIV-1 p24 antigen production in PBMC was calculated. The concentration that resulted in a 50% reduction in p24 antigen expression (EC_50_) was determined from the dose-response curve.

### 3.7. Inhibition of HIV-1 p24 Antigen Production in Chronically Infected Cells

Chronically infected cell with HIV-1_IIIB_ (H9/HIV-1_IIIB_) were washed three times with PBS to remove free virus particle. 200 µL/well (3 × 10^5^ cells/mL) of the cell suspension was cultured for three days in a 96-well culture plate with different concentrations of compound. The p24 antigen in the culture supernatants was assayed by ELISA.

### 3.8. Co-Cultivation Assay

Cell-to-cell fusion between normal C8166 cells and H9 cells chronically infected withHIV-1_IIIB_ was quantified under an inverted microscope. First, 3 × 10^4^ C8166 cells co-cultured with 1 × 10^4^ H9/HIV-1_IIIB_ cells in the presence or absence of the compound at varying concentrations. They were incubated at 37 °C in a humidified atmosphere of 5% CO_2_. Dextran sulfate (DS) was used as positive control. After 6 h incubation, the number of syncytia was scored under an inverted microscope [17]. 

### 3.9. Inhibition Assay of Recombinant HIV-1 RT Activity

HIV-1 reverse transcriptase (RT) activity was measured by the ELISA RT kit using a commercially available kit (Roche, Mannheim, Germany) according to the instructions of the manufacturer. The compounds were incubated with DIG-labeled reaction mixture at 37 °C for 2 h, then anti-DIG-POD solution was added, followed by substrate ABTS. Foscarnet was used as a positive control. The absorbency at 405 nm/490 nm was read on the Bio-Tek ELx 800 ELISA reader [17].

### 3.10. Inhibition Assay of Recombinant HIV-1 Protease Activity

The recombinant HIV-1 protease (PR) was expressed and purified, as previously described [18]. HIV-1 PR was diluted in reaction buffer. The compounds were added and incubated for 30 min at room temperature. Fluorescent substrate DABCYL-γ-Abu-Ser-Gln-Asn-Tyr-Pro-Ile-Val-Gln-EDANS (Anaspec, San Jose, USA) was added to initiate the reaction. The mixture was allowed to react for 90 min and the change of the fluorescent signal was monitored. Negative (double distilled water) and positive (1 μM indinavir) controls were included. The percent inhibition of PR activity was calculated.

### 3.11. Binding Potency of the Compound to HIV-1 Integrase

The interaction between the compound and HIV-1 integrase (IN) was determined by SPR using a BIAcore 3000TM biosensor system as previously described [19]. Integrase was immobilized on the surface of the chip. To allow association, various concentrations of the compound diluted in HBS-EP was applied to a chip containing immobilized integrase. Dissociation of the compound from the integrase was monitored in real-time after application of buffer to wash the chip and the kinetic rate constants for dissociation (*K_d_*) was obtained by fitting the real-time data using BIA evaluation software.

## 4. Conclusions

7-Hydroxymethylcamptothecin showed more potent anti-HIV activity than camptothecin, while 10-hydroxycamptothecin had less efficient anti-HIV activity than camptothecin. The modification of -OH residues at the C-7 site of camptothecin enhanced the antiviral activity, while the modification of -OH residues at the C-10 site of camptothecin decreased the antiviral activity. This study provides the preliminary modification strategy for anti-viral activities enhancement of this compound, though the antiviral mechanistic details need to be further investigated.

## Figures and Tables

**Table 1:** Anti-HIV activities of compounds **1**–**3**. ^a^

Compounds	Cells	HIV strains or enzymes	Assays	EC50 b (ng/mL)	CC50 c (ng/mL)	TId
**1**	C8166	HIV-1_IIIB_	Syncytia	5.7 ± 0.1	137.8 ± 7.8	24.2
		HIV-2_CBL-20_	Syncytia	4.0 ± 0.3		34.5
		HIV-2_ROD_	Syncytia	6.7 ± 0.1		20.6
	PBMC	HIV-1_KM018_	p24	7.4 ± 1.1	75.9 ± 13.1	10.3
**2**	C8166	HIV-1_IIIB_	Syncytia	33.4 ± 0.3	140.9 ± 9.7	4.2
		HIV-2_CBL-20_	Syncytia	13.2 ± 1.0		10.7
		HIV-2_ROD_	Syncytia	25.0 ± 0.9		5.6
	PBMC	HIV-1_KM018_	p24	10.5 ± 0.4	36.3 ± 11.8	3.5
**3**	C8166	HIV-1_IIIB_	Syncytia	0.8 ± 0.2	158.5 ± 13.7	198.1
		HIV-2_CBL-20_	Syncytia	0.5 ± 0.1		317.0
		HIV-2_ROD_	Syncytia	2.4 ± 0.2		66.0
	PBMC	HIV-1_KM018_	p24	0.9 ± 0.1	60.0 ± 10.1	66.7
	C8166	HIV-1_IIIB_/H9	Co-cultivation	> 2000		
	H9/HIV-1_IIIB_	Reverse transcriptase	ELISA	> 17000		
	-	Protease	Fluorescent	> 40000		
	-	Integrase	BIAcore	24570(K_d_)^e^		
	-	Reverse transcriptase	ELISA	> 17000		
	H9 H9/HIV-1_IIIB_ Jurkat Jurkat/HIV-1_IIIB_				18.2 ± 3.2 15.7 ± 2.6 24.4 ± 2.1 41.0 ± 7.0	
AZT	C8166	HIV-1_IIIB_	Syncytia	3.0±0.3	1.12(mg/ml)	
	PBMC	HIV-1_KM018_	p24	2.4±0.3	0.45(mg/ml	

^a^ The data shown in the table are representatives of three independent experiments; ^b^ EC_50_ is the effective concentration that inhibits 50% of viral production; ^c^ CC_50_ is the cytotoxic concentration that reduces 50% of viable cells; ^d^ TI is therapeutic index, and it is ratio of EC_50_ to CC_50_; ^e^ *K_d_* is the kinetic rate constant for dissociation.

## Supplementary Materials

Supplementary File 1
