# Supplementary material for: The Anti-HIV Actions of 7- and 10-Substituted Camptothecins
Source: Molecules. 2009 Dec 31;15(1):138–48. doi: 10.3390/molecules15010138 (PMC6256925; doi:10.3390/molecules15010138)
Supplement: Supplementary File 1 [file molecules-15-00138-s001.pdf]

Molecules **2010**, *15*, 149; doi:10.3390/molecules15010149

OPEN ACCESS

molecules

ISSN 1420-3049

www.mdpi.com/journal/molecules

Correction

Chen, Zheng, *et al*. Anti-HIV Actions of 7- and 10-Substituted Camptothecins. *Molecules*, 2010, *15*, 138-148

Yong-Tang Zheng

Key Laboratory of Animal Models and Human Disease Mechanisms of Chinese Academy of Sciences & Yunnan Province, Kunming Institute of Zoology, Chinese Academy of Sciences, Kunming, Yunnan 650223, China

Received: 3 January 2010 / Published: 4 January 2010

The author list, affiliations and contact information of this paper [1] are revised to read as follows:

Yu-Ye Li [1,2,3,†], Ying-Qian Liu [4,†], Liu-Meng Yang [1], Rui-Rui Wang [1], Wei Pang [1], Shi-Wu Chen [4] Xuan Tian [4,*] and Yong-Tang Zheng [1,*]

[1] Key Laboratory of Animal Models and Human Disease Mechanisms of Chinese Academy of Sciences & Yunnan Province, Kunming Institute of Zoology, Chinese Academy of Sciences, Kunming, Yunnan 650223, China

[2] Graduate School of the Chinese Academy of Sciences, Beijing 100039, China

[3] The First Affiliated Hospital of Kunming Medical College, Kunming, Yunnan 650032, China

[4] State Key Laboratory of Applied Organic Chemistry, Lanzhou University, Lanzhou 730000, China

† These authors contributed equally to this work.

* Authors to whom correspondence should be addressed; E-Mail: zhengyt@mail.kiz.ac.cn (Y-T.Z.); xuant@lzu.edu.cn (X.T.); Tel./Fax: +86 871 5195684 (Y.-T.Z).

References

1. Li, Y.-Y.; Chen, S.-W.; Yang, L.-M.; Wang, R.-R.; Pang, W.; Zheng, Y.-T. The Anti-HIV Actions of 7- and 10-Substituted Camptothecins. *Molecules* **2010**, *15*, 138-148.

© 2010 by the authors; licensee Molecular Diversity Preservation International, Basel, Switzerland. This article is an open-access article distributed under the terms and conditions of the Creative Commons Attribution license (http://creativecommons.org/licenses/by/3.0/).
